# Closing Difficult Laparostomies With the Aid of Botulinum Toxin A: An Audit of 12 Cases

**DOI:** 10.7759/cureus.14066

**Published:** 2021-03-23

**Authors:** Jason R Laurens, Amanda Foster, Andrew Hardley

**Affiliations:** 1 General Surgery, Fiona Stanley and Fremantle Hospital Group, Perth, AUS; 2 General Surgery, Joondalup Health Campus, Perth, AUS

**Keywords:** laparostomy, open abdomen, damage control laparotomy, temporary abdominal closure, botulinum toxin a, primary fascial closure.

## Abstract

Background

Obtaining primary fascial closure following laparostomy can be difficult; especially with fascial retraction or large pre-existing fascial defects. Various techniques have been described in the literature which attempt to improve reapproximation rates. Most techniques described comprise the use of adjuncts including Bogota Bags, negative pressure dressings, anchor devices and various types of mesh. While most techniques achieve primary closure, less achieve primary fascial closure. Botulinum toxin A (BTA) has proven a beneficial adjunct in repairing large ventral herniae. While there is limited research in the use of BTA in the acute setting of laparostomy closure its benefits in elective repair may prove transferrable with the appropriate protocols.

Method

This retrospective study reviewed 12 cases where BTA was used as an adjunct to close laparostomy. It compared primary fascial closure rates to historical controls at the same institution.

Results

Seven males and five females. Median age 63.5 years. Median BMI 32.95. Median days from BTA injection to primary fascial closure 9.5. Median 18 days from primary operation to primary fascial closure. 83% of patients achieved primary fascial closure with the rest achieving partial closure with the residual defect bridged with biological mesh. At the time of review, there was only one resulting ventral hernia in a patient with a BMI of 51.7 at the time of surgery.

Conclusion

While BTA does not guarantee primary fascial closure in laparostomy this study would indicate it improves primary fascial closure rates and can be added to any other existing method for managing the open abdomen. As BTA can be injected via the open abdomen or with ultrasound guidance it can be performed by any appropriately trained surgeon, anaesthetist or radiologist making its use widely achievable. Retrospectively registered.

## Introduction

Laparostomy, damage control laparotomy (DCL) and open abdomen are relatively interchangeable terms describing techniques in which the abdomen is deliberately left open following emergency surgery. The concept of laparostomy was first described by Pringle [1] and Halsted [2] in their respective papers in 1908 and 1913. Since their initial articles were published, there have been numerous papers on the indications for leaving the abdomen open, safely containing the abdominal contents and techniques for closing the open abdomen.

While Pringle and Halsted both described the open abdomen in the setting of treating haemorrhage, the list of indications has increased. Coccolini et al produced the World Society for Emergency Surgery guidelines on the open abdomen, describing trauma, peritonitis, vascular emergencies, and pancreatitis where there is high risk of abdominal compartment syndrome as indications for leaving the abdomen open [3]. While there is debate as to the efficacy of laparostomy in improving patient outcomes in an abdomen that could otherwise be closed during initial laparotomy [4], there are times when laparostomy is necessary, such as those whose abdomens cannot be closed due to poor condition of the fascia, extreme visceral swelling or when reoperation is planned. Temporary abdominal closure (TAC) describes the method for providing protection of the abdominal organs during the period of laparostomy. Considering both physiological factors and the severity of injury, TAC along with appropriate intensive care support has been shown to reduce mortality, leading to the increased use of temporary laparostomy as part of a damage control approach [5]. Having decided to leave the abdomen open, the surgeon is faced with several specific challenges. The ideal temporary closure prevents evisceration, allows control of fluid losses, allows ready access to the abdominal cavity for re-look laparotomy and preserves both fascial structure and length [6]. A wide variety of techniques can be used for temporary closure, from simple packing to complex vacuum-assisted dressings and fascial tension meshes and devices. One of the major barriers to successful closure of the open abdomen is retraction of the fascia, a state caused by unopposed contraction of the lateral abdominal wall muscles. Several methods have been designed to decrease retraction, ranging from vacuum-assisted dressings [7] to sequential tightening of an inlay mesh, such as the Wittmann patch technique [8,9]. These are all designed to provide countertraction to the abdominal wall muscles, allowing sequential closure of the fascial defect. Nevertheless, in many cases, the fascial defect remains too large and the patient is left with a planned ventral hernia, requiring delayed repair.

Botulinum toxin A (BTA, Botox®, Allergan, Inc, Irvine, CA) is a neuromodulator that binds to glycoproteins in the cholinergic pathway, temporarily blocking both motoric and autonomic innervation. Dressler reports that the effect of BTA can be detected after two days, with maximal effect at two weeks and gradually declining over 2.5 months [10]. The first prospective study using BTA to close abdominal wall hernia was undertaken in 2009 by Ibarra-Hurtado et al, who injected BTA into 12 study patients bilaterally. The decrease in defect size was measured prior to BTA and followed with sequential CT until hernia repair achieved primary fascial closure at four weeks in all 12 patients. A mean decrease in defect of 5.25 cm was seen [11]. Further studies discuss how the application of BTA increases lateral abdominal wall muscle length, therefore reducing defect size allowing abdominal wall reconstruction with fascial approximation [12-14]. Fascial approximation after chemical component separation has been reported between 83.5% and 100% in both open and laparoscopic repair of complex ventral hernia [12,15,16].

While there is limited literature that describes the use of BTA to aide primary fascial closure in laparostomy [17], its application is readily transferable from elective ventral hernia repair to the emergency setting. In both cases, BTA is used to overcome the challenge of fascial retraction, with the resulting “chemical component separation” allowing bridging of large defects not otherwise closable. The ease of BTA injection by trained personnel including surgeons, anaesthetists, and radiologists either during surgery or as a stand-alone procedure makes its administration possible across large and small facilities.

This article was previously accepted as a meeting abstract at the RACS Annual Scientific Congress on the 12th of May 2020 and published as an abstract in the ANZ Journal of Surgery on the 4th of June 2020.

## Materials and methods

Patients were initially identified by retrospectively reviewing pharmacy records for all patients prescribed BTA by a general surgeon across the Fiona Stanley/Fremantle Hospital group between January 2015 and December 2018. The 29 identified patients’ medical records were examined, identifying 12 patients who had an emergency laparostomy and injection of BTA into their lateral abdominal wall muscles during their care. The remaining 17 patients prescribed BTA by a general surgeon underwent either UGI or anorectal procedures.

**Figure 1:**
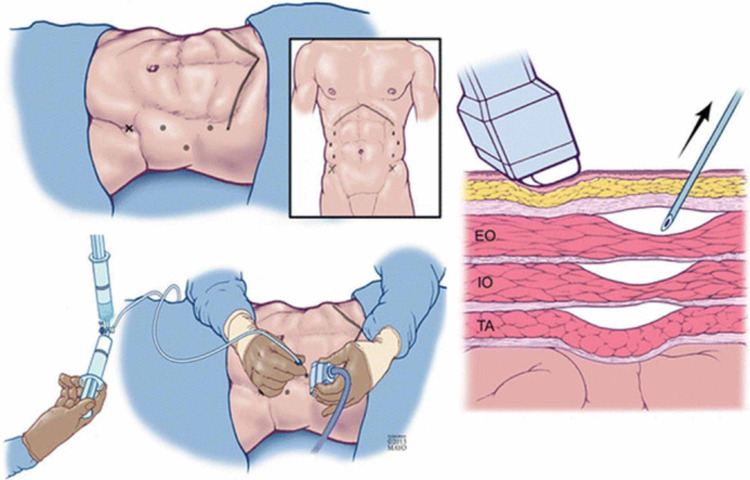
Application of botulinum toxin A. EO, the external abdominal oblique muscle; IO, the internal abdominal oblique muscle; TA, the transversus abdominus muscle. Reproduced from Zendejas et al: Outcomes of chemical component paralysis using botulinum toxin for incisional hernia repairs: World J Surg. 2013; 37:2830-2837 with permission from Springer. Available via license: CC BY-NC-ND 4.0

Our technique for BTA was constant, with our standard approach beginning with the identification of the three lateral abdominal wall muscles (external oblique, internal oblique and transversus abdominis) using ultrasound. BTA was then injected into all three muscles, with 50 IU injected at each of three separate points on each side of the abdominal wall like that described by Zielinski et al [17]. A total dose of BTA 300 IU is given per patient (Figure 1).

Demographic and comorbidity data points were collected, including sex, age, BMI, smoking status, presence of diabetes, cardiovascular disease, respiratory disease and liver dysfunction or irregular clotting profiles. Surgical details were collected, including date of primary operation, date of BTA injection, date of fascial closure, adjuncts used to aid primary closure and resulting hernia.

The cases selected for the use of BTA underwent comparison against a previous audit of all (33) laparostomies undertaken at the same centre from 2010 to 2017, which did not involve the use of BTA. No adjustment was made in order to validate the comparison due to the low number of cases.

## Results

Between January 2015 and December 2018 12 cases were identified which met inclusion criteria. We used BTA on a selective basis, for patients predicted to be unlikely to be closable primarily after temporary laparostomy. Seven male and five females. They were predominantly older patients (median age 63.5 (40-79)), with all but two patients being overweight or obese (median BMI 33.2 (18.3-51.7)).

Obesity, smoking, diabetes, cardiorespiratory disease and liver disease are known risk factors for incisional hernia development. As seen in Table 1, all but two patients (83%) had at least one of these risk factors, with half having two or more risk factors present.

**Table 1:** Patient demographics. T2DM: type 2 diabetes mellitus; CVD: cardiovascular disease; BMI: body mass index.

Case	Sex	Age	BMI	Smoker	Diabetic	CVD	Respiratory disease	Liver disease/clotting factors
1	M	62	36.4	No	T2DM	No	Asthma	No
2	F	40	51.7	No	No	No	No	No
3	M	51	18.3	Yes	T2DM	No	No	No
4	F	66	45.6	No	T2DM	No	No	No
5	M	79	35.6	Ex-Smoker	No	No	No	No
6	F	78	30.3	No	No	No	No	No
7	M	62	40.1	Yes	No	No	No	Polycythemia vera
8	F	53	19	No	No	No	Asthma	No
9	M	49	26.9	No	No	No	No	No
10	M	65	29.4	No	No	No	No	No
11	M	78	29.9	Ex-smoker	No	PVD	No	No
12	F	79	35.6	No	T2DM	No	No	No
	M = 7; F = 5	Median 63.5	Median 32.9					

Our most common indication for laparostomy was bowel obstruction, with requirement for re-look laparotomy. Half of our patients had an existing ventral hernia prior to initial laparotomy. BTA in all cases was delayed, being performed either at a subsequent re-look laparotomy or as a stand-alone procedure. All patients had multiple adjuncts to facilitate closure in addition to BTA. In almost all cases, BTA was added to a combination of vacuum-assisted closure and sequentially tightened prolene bridging mesh. A median of 7 procedures was required to close the abdomen in the patients (range 4-11). Primary fascial closure was achieved in 10 patients, while the remaining two achieved primary skin closure with partial fascial closure with inlay biological mesh, Biodesign® (Cook Medical, Bloomington, IN) and GORE® BIO-A® (W.L Gore & Associates, Flagstaff, AZ) each used once to bridge the remaining defect. Median time from BTA to closure was 9.5 days, with a median overall time to closure of 18 days. This lengthy time and high median number of procedures to closure fits with our practice of using BTA selectively as an adjunct only in those most difficult cases where the wide fascial defect predicts failure of primary closure (Table 2).

**Table 2:** Surgical indications, adjuncts, and outcomes. *Degree of contamination*: 1 Clean, 2 Clean/contaminated, 3 Contaminated, 4 Dirty. *Indication for laparostomy*: 1 Severe physiological derangement, 2 Persistent source of peritonitis, 3 Planned re-look for intestinal ischaemia/remove packs, 4 Significant wall defects. *Timing of BTA*: 1 During re-look without closure, 2 Stand-alone procedure in theatre/ICU. *Adjuncts in treatment*: 1 BTA injection, 2 Abdominal Vac Dressing i.e., Abthera™, 3 Fascial traction sutures, 4 Bridging mesh – removed at closure, 5 Permanent mesh (Biologic).

Case	Indication for surgery	Contamination	Indication for laparostomy	Timing of BTA	Laparostomy to BTA (days)	Total number of procedures to close	BTA to close (days)	Total days open abdomen	Adjuncts in closure	Primary fascial closure	Resulting hernia
1	Perforated viscous within ventral hernia	3	1 + 2	1	21	11	14	38	1,2,4	Yes	No
2	Ischaemic bowel	4	1 + 2	2	3	5	5	8	1,2,4	Yes	Yes
3	Anastomotic leak	3	1 + 2	1	28	11	14	42	1,2,4	Yes	No
4	Perforated viscous within ventral hernia	4	2 + 4	1	8	7	11	19	1,2,4	Yes	No
5	SBO within ventral hernia	3	3 + 4	1	27	8	6	33	1,2,3,4,5	No	No
6	Blunt trauma	2	1 + 3	2	2	5	8	10	1,2	Yes	Unknown
7	SBO within ventral hernia	1	3 + 4	1	4	8	19	23	1,2,4,5	No	No
8	Vascular emergency	2	3	1	4	8	13	17	1,2,4	Yes	No
9	Ischaemic bowel	4	3	1	6	5	8	14	1,2,4	Yes	No
10	Perforated SBO within ventral hernia	2	2	2	0	4	4	4	1,2,4,5	Yes	No
11	LOB within ventral hernia	1	4	1	3	6	18	21	1,2,4	Yes	No
12	SBO	1	1 + 4	1	2	5	7	10	1,2,4	Yes	No
					Median 4	Median 6.5	Median 9.5	Median 18		10/12	1/12

Data from the previous laparostomy audit were examined and compared (Tables 3, 4).

**Table 3:** Comparative results between BTA and non-BTA cases. BTA: Botulinum toxin A.

	BTA, N=12	Non-BTA, N=33
Pre-existing ventral hernia	6 (50%)	10 (30.3%)
Median No. operations to close	6.5 (4-11)	3.3 (1-9)
Median No. days abdomen open	18 (4-42)	3 (0-30)
Primary fascial closure	10 (83%)	23 (70%)

**Table 4:** Methods of closure for comparative audit cases.

Abthera^™^	25
Fascial traction + Abthera^™^	2
3M^™^ IOBAN^™^ dressing	2
Skin closure	1
Bogota bag	1
Not stated	2

## Discussion

This article is one of the first to describe the use of BTA as an adjunct in the setting of emergency laparostomy. A previous audit of all laparostomies undertaken at our centre from 2010 to 2017 identified 33 cases; of these 23 achieved primary fascial closure using the same techniques without BTA. The addition of BTA as an adjunct improved primary fascial closure rates by the same group of surgeons using similar techniques to close the open abdomen from 70% to 83%. In addition, this control includes all laparostomies, whereas our practice is to reserve the use of BTA for the most difficult abdominal closures, where we predict failure by conventional methods. This means our higher closure rate with BTA occurs despite this subgroup being the most likely group to fail.

Multiple other methods have been described to prevent fascial retraction and facilitate closure of the open abdomen. One of the earliest methods described by Steinberg in 1973 discussed leaving the abdomen open for 48 to 72 hours following the initial operation in the management of acute suppurative peritonitis, using a 4-inch gauze pack placed within the peritoneal cavity to provide sterile coverage of the organs while untied 0 wires placed over the gauze held it in place without closing the peritoneal cavity [18]. Khasawneh and Zielinski reviewed methods for TAC regarding assisting primary fascial closure; they concluded that no method described was ideal [19], although they reference Burlew et al who report 100% fascial approximation can be achieved with a sequential vacuum-assisted dressing protocol [20]. Whilst Burlew et al report 100% closure in the 29 patients who followed their protocol, of the 22 patients who did not follow their protocol they achieved fascial closure in only 55% [20]. Coccolini et al report overall primary fascial closure in 64.7% of patients, the lowest recorded rate was 59.9% with Negative Pressure Wound Therapy while their highest rate of primary fascial closure was 71.3% using the Bogota bag + skin closure technique [21]. Use of the Abdominal Reapproximation Anchor Device has been shown to have primary fascial closure rates between 61% and 88% [22]. The results of this study show favourable outcomes when BTA is administered as an adjunct to conventional closure methods. Coccolini et al [21] and Okullo et all [22] who have investigated numerous methods show lower rates of primary fascial closure.

One of the benefits of BTA is that it can be added to any of the above methods. The chemical component separation it produces allows relaxation and elongation of the abdominal wall muscles, while preserving their structural integrity for any future abdominal wall reconstruction. The limitation is obviously that BTA takes several days to become therapeutic [10], meaning it should ideally be injected early in patients where substantial oedema or pre-existing fascial defects mean closure is expected to be a sequential affair over multiple laparostomies. As BTA can be injected during laparostomy or as a standalone procedure it implores further teaching of surgeons, anaesthetists, and radiologists to improve access to this adjunct. Improved refinement of the use of BTA may lead to improved primary fascial closure, in turn likely reducing ventral hernia or use of permanent bridging mesh, which comes with its own complications.

The limitation of this study is the relatively low number of cases that have been undertaken. Due to the nature of trauma and emergency surgery, and the extreme variables associated with the patient demographics it may also be hard to clearly define which patients will benefit from the use of BTA in their management. Further comparison of the previous audit to this case series highlighted the degree of contamination and preceding ventral hernia as possible indicators; this was due to the number of relooks required and progressive closure of the large abdominal wall defects leading to prolonged open abdomen. Further cases would need to be compared to give a stronger picture of identifiable indicators.

## Conclusions

While there are many published studies investigating the various techniques used to close laparostomy, there are few which include the use of BTA as an adjunct in the acute setting. This review showed primary fascial closure in 83% of patients who had BTA therapy in conjunction with more traditional methods. This improves upon our previous rate of 70% primary fascial closure despite the BTA group comprising a sub selected poor prognosis group. If BTA can be injected at the first available opportunity it will likely aide timely fascial approximation due to its ability to lengthen lateral abdominal wall muscles reducing the laparostomy defect. While this review centred on primary fascial closure, resulting ventral hernia should also be a driving force in improving fascial closure rates. The use of BTA as an adjunct means the laparostomy closure will be under less lateral tension for approximately 2.5 months, this may reduce the incidence of ventral hernia through incomplete fascial closures. The ease of BTA application as an adjunct to many of the techniques already used by surgeons will likely improve primary fascial closure rates.
